# Prognostic value of microRNAs in pancreatic cancer: a meta-analysis

**DOI:** 10.18632/aging.103214

**Published:** 2020-05-18

**Authors:** Fei Zhao, Chao Wei, Meng-Ying Cui, Qiang-Qiang Xia, Shuai-Bin Wang, Yue Zhang

**Affiliations:** 1, Department of Traditional Chinese Medicine, The First Affiliated Hospital of Shandong First Medical University, Jinan, China; 2 College of Integrated Traditional Chinese and Western Medicine, Jining Medical University, Jining, Shandong, China; 3 Department of Hepatobiliary and Pancreatic Surgery, The Second Hospital of Jilin University, Changchun, Jilin, China; 4 Department of Neurobiology, University of Alabama at Birmingham, Birmingham, AL 35233, USA; 5 Department of Urology, The Second Affiliated Hospital of Wenzhou Medical University, Wenzhou, Zhejiang, China; 6 Department of Genetics, University of Alabama at Birmingham, Birmingham, AL 35233, USA; 7 Comprehensive Cancer Center, University of Alabama at Birmingham, Birmingham, AL 35233, USA

**Keywords:** microRNA, pancreatic cancer, prognosis, meta-analysis

## Abstract

Background: The prognostic impact of microRNA (miRNA) expression levels in pancreatic cancer (PC) has been estimated for years, but the outcomes are controversial and heterogeneous. Therefore, we comprehensively reviewed the evidence collected on miRNA expression in PC to determine this effect.

Results: PC patients with high miR-21 (HR=2.61, 95%CI=1.68-4.04), miR-451a (HR=2.23, 95%CI=1.23-4.04) or miR-1290 (HR=1.43, 95%CI=1.04-1.95) levels in blood had significantly poorer OS (P<0.05). Furthermore, PC patients with high miR-10b (HR=1.73, 95%CI=1.09-2.76), miR-17-5p (HR=1.91, 95%CI=1.30-2.80), miR-21 (HR=1.90, 95%CI=1.61-2.25), miR-23a (HR=2.18, 95%CI=1.52-3.13), miR-155 (HR=2.22, 95%CI=1.27-3.88), miR-203 (HR=1.65, 95%CI=1.14-2.40), miR-221 (HR=1.72, 95%CI=1.08-2.74), miR-222 levels (HR=1.72, 95%CI=1.02-2.91) or low miR-29c (HR=1.39, 95%CI=1.08-1.79), miR-126 (HR=1.55, 95%CI=1.23-1.95), miR-218 (HR=2.62, 95%CI=1.41-4.88) levels in tissues had significantly shorter OS (P<0.05).

Conclusions: In summary, blood miR-21, miR-451a, miR-1290 and tissue miR-10b, miR-17-5p, miR-21, miR-23a, miR-29c, miR-126, miR-155, miR-203, miR-218, miR-221, miR-222 had significant prognostic value.

Methods: We searched PubMed, EMBASE, Web of Science and Cochrane Database of Systematic Reviews to recognize eligible studies, and 57 studies comprising 5445 PC patients and 15 miRNAs were included to evaluate the associations between miRNA expression levels and overall survival (OS) up to June 1, 2019. Summary hazard ratios (HR) with 95% confidence intervals (CI) were calculated to assess the effect.

## INTRODUCTION

Much effort has been made over a long period of time to identify prognostic biomarkers in pancreatic cancer (PC) patients. Fortunately, a large body of literature has covered the survival of PC patients with abnormal microRNA (miRNA) expression [1–169]. Among all kinds of human cancers, PC has one of the worst prognoses, with a 5-year overall survival (OS) rate of lower than 5% [170]. Despite advances in clinical treatments and new surgical techniques, the survival rate of PC patients has been low for more than 30 years [171]. PC is highly aggressive; therefore, distant metastasis and tissue invasion may occur at early stages [172]. Since invasion and metastasis are the biggest obstacles to effective treatment of PC, it is imperative to explore the molecular biological mechanism leading to such invasive behavior to improve the survival time of patients.

miRNAs are small noncoding RNAs involved in gene regulation [173]. In cancers, a few upregulated miRNAs can serve as oncogenes (oncomiRs) [174], and downregulated miRNAs can serve as tumor suppressors [175]. Expression profiling data analyses have revealed signatures of diagnosis and prognosis that have been employed to stratify various tumor types [174, 176]. As a consequence, miRNAs have the potential to turn into clinical biomarkers for human tumors and into molecular therapeutic targets [177].

Despite comprehensive studies focused on illustrating the molecular biological mechanisms in PC, there are still challenges confronting the identification of minimally invasive and sensitive biomarkers of prognosis. Consequently, it is of vital significance to find prognostic signatures that can be conveniently and reliably applied in the clinical setting to improve the survival time of PC patients.

Increasing evidence indicates that miRNAs have the potential to act as PC prognostic biomarkers in clinical practice [1–169]. Regrettably, there has not been a meta-analysis to evaluate the relationship between dysregulated miRNA expression and survival in PC patients. In view of our previous work, meta-analyses of miRNA expression and cancer patients [178, 179], it is necessary to conduct the current work by searching the recently published literature about miRNAs as prognostic tools in PC tissue or blood.

## RESULTS

### Meta-analysis

**Figure 1:**
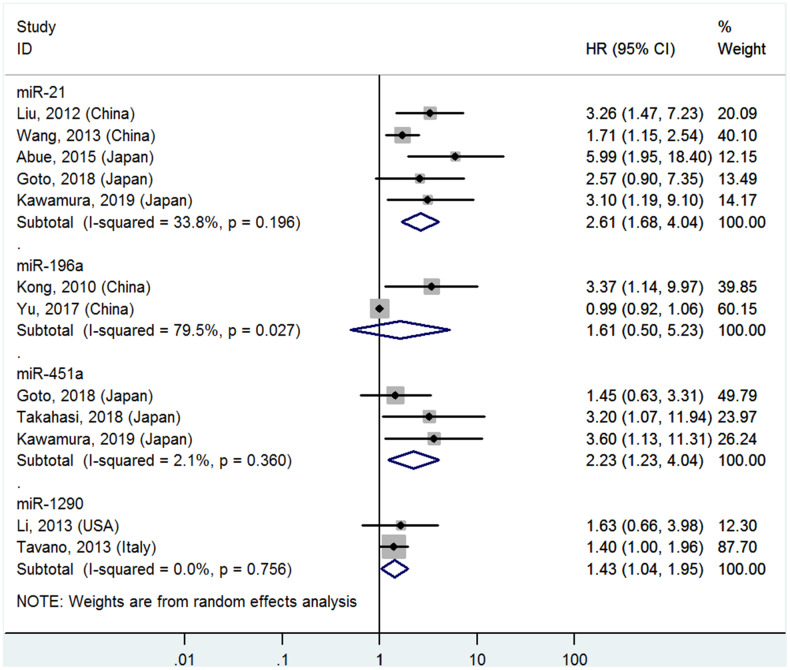
**Forest plot about OS of PC patients with high miR-21, miR-196a, miR-451a or miR-1290 level in blood**

**Figure 2:**
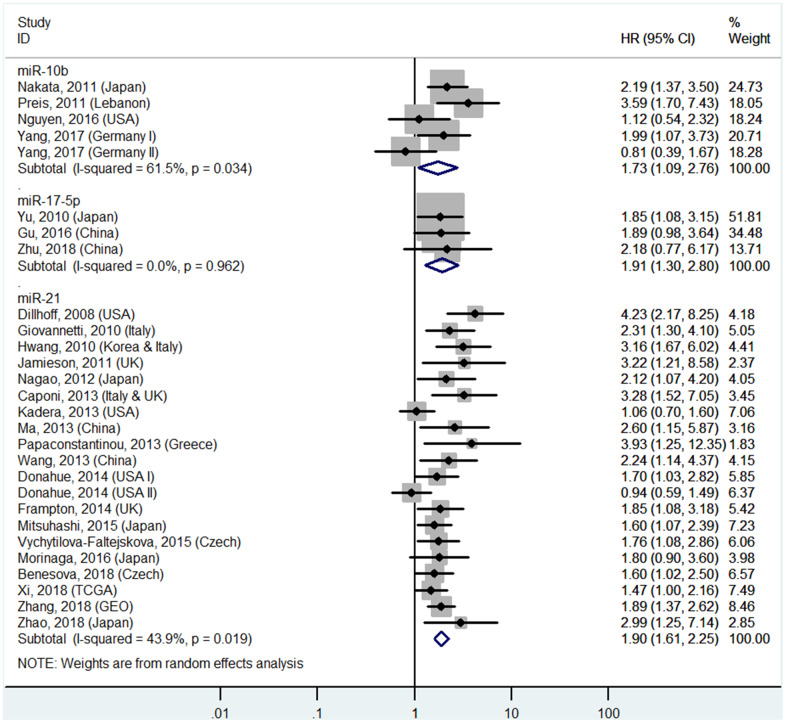
**Forest plot about OS of PC patients with high miR-10b, miR-17-5P or miR-21 level in tissue.**

**Figure 3:**
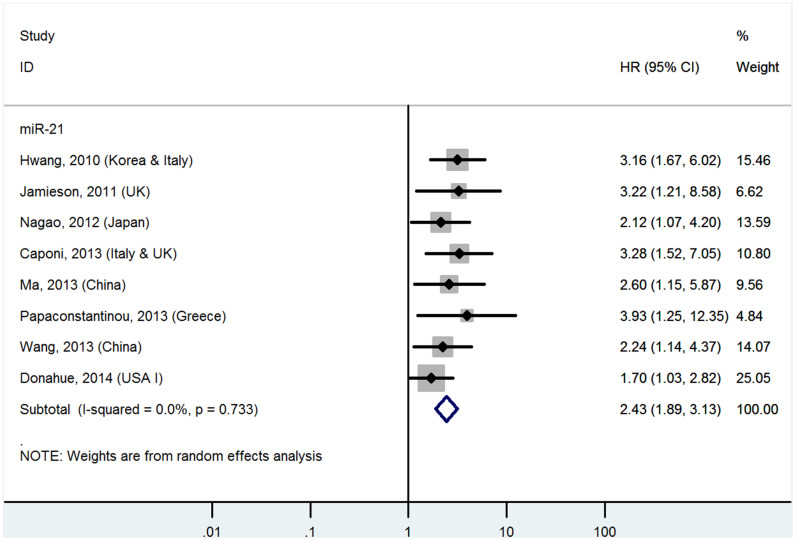
**Forest plot about OS of PC patients with high miR-21 level in tissue (multivariate analysis).**

**Figure 4:**
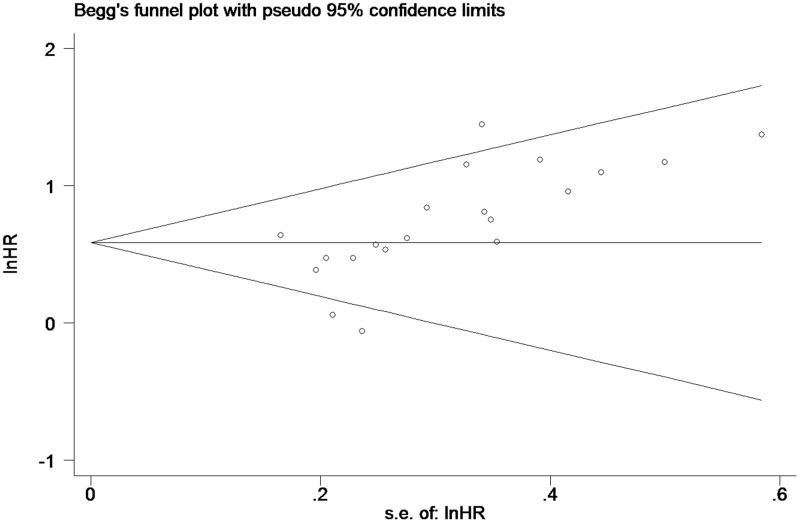
**Begg’s funnel plot about OS of PC patients with high miR-21 level in tissue.**

**Figure 5:**
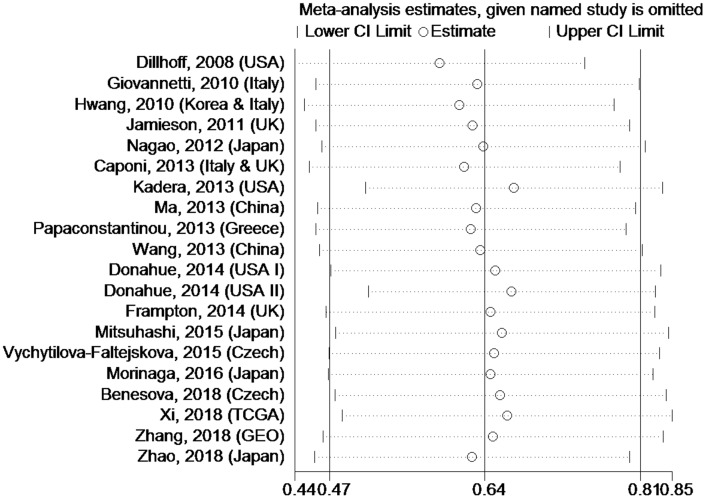
**Sensitivity analysis about OS of PC patients with high miR-21 level in tissue.**

**Figure 6:**
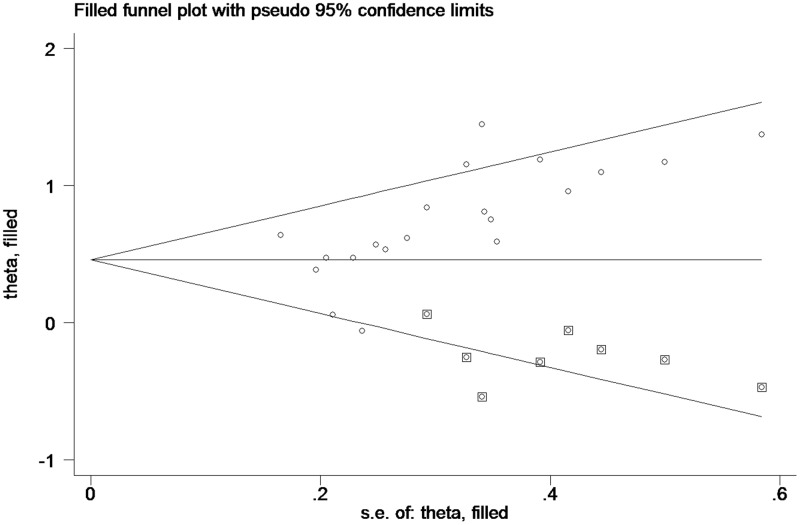
**Funnel plot about pooled analysis adjusted with trim and fill method of OS of PC patients with high miR-21 level in tissue. Circles: included studies; diamonds: presumed missing studies.**

**Figure 7:**
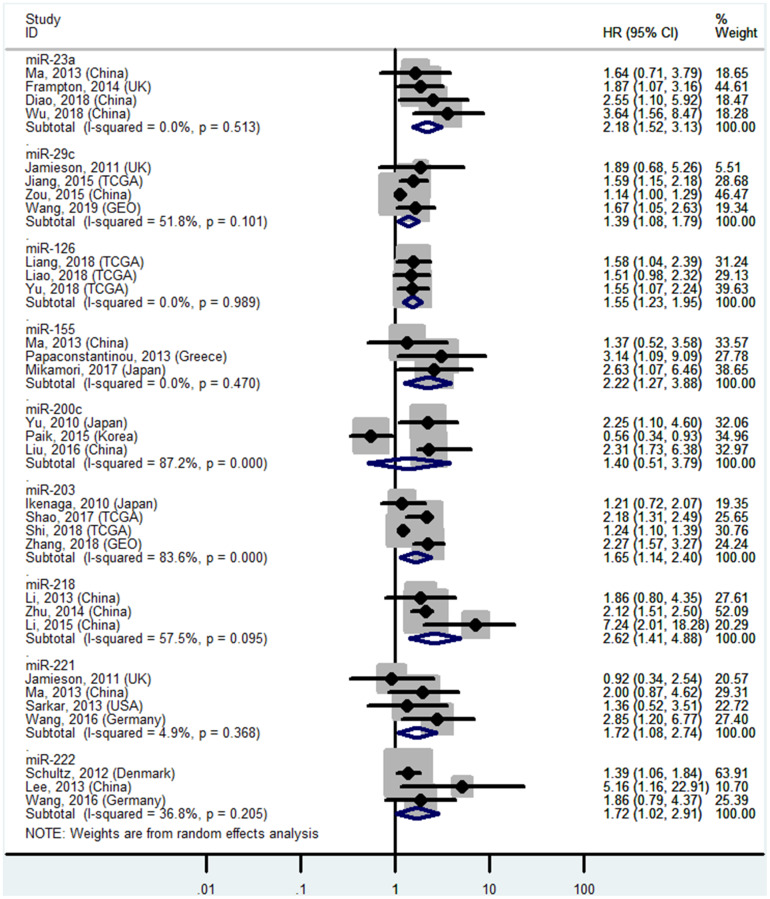
**Forest plot about OS of PC patients with high miR-23a, miR-155, miR-203, miR-221, miR-222 or low miR-29c, miR-126, miR-200c, miR-218 level in tissue.**

An overview of the HR with 95%CI obtained from the overall comprehensive analysis for all included miRNAs is shown in Table 1. Based on the logical order of the miRNA names, the forest plot, Begg’s funnel plot, sensitivity analysis and funnel plot of the merged analysis adjusted with the trim and fill method are shown in Figures 1–7. The mean NOS score of the included studies was 7.0 (5.0-8.0), indicating that their quality was adequate (Table 2).

**Table 1:** Summary about results of meta-analysis for miRNA expression in pancreatic cancer.

**miRNA**	**Sample**	**Survival analysis**	**Number of articles**	**Included studies**	**HR**	**95%CI**	**Figure**	**P value**	**Heterogeneity (Higgins I^2^ statistic)**	**Total patients**
High miR-21	Blood	OS	5	4-8	2.61	1.68-4.04	2	<0.01	I^2^=33.8%, P=0.20	326
High miR-196a	Blood	OS	2	16,17	1.61	0.50-5.23	2	0.43	I^2^=79.5%, P=0.03	66
High miR-451a	Blood	OS	3	7,8,23	2.23	1.23-4.04	2	<0.01	I^2^=2.1%, P=0.36	137
High miR-1290	Blood	OS	2	24,26	1.43	1.04-1.95	2	0.03	I^2^=0.0%, P=0.76	223
High miR-10b	Tissue	OS	4	35-38	1.73	1.09-2.76	3	0.02	I^2^=61.5%, P=0.03	375
High miR-17-5p	Tissue	OS	3	39-41	1.91	1.30-2.80	3	<0.01	I^2^=0.0%, P=0.96	164
High miR-21	Tissue	OS	19	5,43-60	1.90	1.61-2.25	3	<0.01	I^2^=43.9%, P=0.02	1947
High miR-21	Tissue	OS^m^	8	5,45-48,50-52	2.43	1.89-3.13	4	<0.01	I^2^=0.0%, P=0.73	592
High miR-21	Tissue	OS^Adjusted^			1.58	1.32-1.89		<0.01	I^2^=58.6%, P<0.01	
High mIR-23a	Tissue	OS	4	50,53,61,62	2.18	1.52-3.13	8	<0.01	I^2^=0.0%, P=0.51	251
Low miR-29c	Tissue	OS	4	33,46,69,70	1.39	1.08-1.79	8	0.01	I^2^=51.8%, P=0.10	463
Low miR-126	Tissue	OS	3	27,68,82	1.55	1.23-1.95	8	<0.01	I^2^=0.0%, P=0.99	455
High miR-155	Tissue	OS	3	14,50,51	2.22	1.27-3.88	8	<0.01	I^2^=0.0%, P=0.47	211
Low mIR-200c	Tissue	OS	3	109-111	1.40	0.51-3.79	8	0.51	I^2^=87.2%, P<0.01	258
High miR-203	Tissue	OS	4	59,112-114	1.65	1.14-2.40	8	<0.01	I^2^=83.6%, P<0.01	619
Low miR-218	Tissue	OS	3	121-123	2.62	1.41-4.88	8	<0.01	I^2^=57.5%, P=0.10	248
High miR-221	Tissue	OS	4	46,50,125,126	1.72	1.08-2.74	8	0.02	I^2^=4.9%, P=0.37	187
High miR-222	Tissue	OS	3	28,126,127	1.72	1.02-2.91	8	0.04	I^2^=36.8%, P=0.21	322

HR: hazard ratios; CI: confidence intervals; OS: overall survival; ^m^multivariate analysis; ^Adjusted^Adjusted with the trim and fill method.

**Table 2:** Newcastle-Ottawa scale quality assessment results.

**First author**	**Year**	**Reference**	**Selection**	**Comparability**	**Outcome**	**Total**
Liu	2012	[4]	★★★	★★	★★	7
Wang	2013	[5]	★★★	★★	★★	7
Abue	2015	[6]	★★★	★★	★★	7
Goto	2018	[7]	★★★	★★	★★	7
Kawamura	2019	[8]	★★★	★★	★★★	8
Mikamori	2017	[14]	★★★	★★	★★★	8
Kong	2010	[16]	★★★	★★	★★	7
Yu	2017	[17]	★★★	★★	★★	7
Takahasi	2018	[23]	★★★	★★	★★	7
Li	2013	[24]	★★★	★★	★★★	8
Tavano	2013	[26]	★★★	★★	★★	7
Liao	2018	[27]	★★★	★★	★★	7
Schultz	2012	[28]	★★★	★★	★★	7
Wang	2019	[33]	★★	★	★★★	6
Nakata	2011	[35]	★★	★	★★★	6
Preis	2011	[36]	★★★	★★	★★	7
Nguyen	2016	[37]	★★★	★★	★★	7
Yang	2017	[38]	★★★	★★	★★★	8
Yu	2010	[39]	★★★	★★	★★★	8
Gu	2016	[40]	★★★	★★	★★	7
Zhu	2018	[41]	★★	★	★★	5
Dillhoff	2008	[43]	★★	★	★★★	6
Giovannetti	2010	[44]	★★★	★★	★★★	8
Hwang	2010	[45]	★★★	★★	★★★	8
Jamieson	2011	[46]	★★★	★★	★★	7
Nagao	2012	[47]	★★★	★★	★★	7
Caponi	2013	[48]	★★★	★★	★★★	8
Kadera	2013	[49]	★★★	★★	★★★	8
Ma	2013	[50]	★★★	★★	★★	7
Papaconstantinou	2013	[51]	★★★	★★	★★★	8
Donahue	2014	[52]	★★★	★★	★★★	8
Frampton	2014	[53]	★★★	★★	★★	7
Mitsuhashi	2015	[54]	★★★	★★	★★	7
Vychytilova-Faltejskova	2015	[55]	★★	★	★★	5
Morinaga	2016	[56]	★★★	★★	★★★	8
Benesova	2018	[57]	★★★	★★	★★	7
Xi	2018	[58]	★★	★	★★★	6
Zhang	2018	[59]	★★	★	★★★	6
Zhao	2018	[60]	★★★	★★	★★★	8
Diao	2018	[61]	★★	★	★★	5
Wu	2018	[62]	★★★	★★	★★	7
Liang	2018	[68]	★★	★	★★★	6
Jiang	2015	[69]	★★	★	★★	5
Zou	2015	[70]	★★★	★★	★★	7
Yu	2018	[82]	★★★	★★	★★★	8
Yu	2010	[109]	★★★	★★	★★★	8
Paik	2015	[110]	★★★	★★	★★★	8
Liu	2016	[111]	★★★	★★	★★★	8
Ikenaga	2010	[112]	★★★	★★	★★★	8
Shao	2017	[113]	★★	★	★★★	6
Shi	2018	[114]	★★	★	★★★	6
Li	2013	[121]	★★★	★★	★★	7
Zhu	2014	[122]	★★★	★★	★★	7
Li	2015	[123]	★★★	★★	★★★	8
Sarkar	2013	[125]	★★	★	★★★	6
Wang	2016	[126]	★★★	★★	★★	7
Lee	2013	[127]	★★★	★★	★★	7

### High miR-21, miR-451a and miR-1290 levels in the blood predict poor OS

Five studies [4–8] analyzed the connections between high blood miR-21 levels and OS, indicating that PC patients with high blood miR-21 levels had significantly poorer OS than those with low levels (HR=2.61, 95%CI=1.68-4.04, P<0.01, Figure 1).

Two studies [16, 17] reported the relationship between high blood miR-196a levels and OS, but no significant associations were found between high blood miR-196a and OS (HR=1.61, 95%CI=0.50-5.23, P=0.43, Figure 1).

Three studies [7, 8, 23] focused on the correlativity between high blood miR-451a levels and OS, indicating that PC patients with high miR-451a levels had significantly shorter OS than those with low levels (HR=2.23, 95%CI=1.23-4.04, P<0.01, Figure 1).

Two studies [24, 26] stressed the pertinence between high blood miR-1290 levels and OS, suggesting that PC patients with high miR-1290 levels had significantly worse OS than those with low levels (HR=1.43, 95%CI=1.04-1.95, P=0.03, Figure 1).

### High miR-10b, miR-17-5P, miR-21, miR-23a, miR-155, miR-203, miR-221, and miR-222 levels or low miR-29c, miR-126, and miR-218 levels in tissues predict poor OS

The details are shown in Table 1 and Figures 2 and 7.

### High miR-21 levels in tissues predict poor OS (multivariate analysis)

The details are shown in Table 1 and Figure 3.

### Publication bias

Begg’s funnel plot was employed to estimate publication bias in the study of OS in PC patients with high tissue miR-21 levels (Figure 4). The results showed that the P value was less than 0.01, indicating the presence of publication bias.

### Sensitivity analysis

Sensitivity analysis was used to estimate whether any single study had undue influence on the OS of PC patients with high tissue miR-21 levels (Figure 5). The outcome showed that no single investigation significantly affected the pooled HR and 95%CI.

### The trim and fill method

As such (Figure 4), the trim and fill method was conducted, and the pooled HR was recalculated with assumed lost studies to assess dissymmetry in the funnel plot (Figure 6), manifesting no publication bias (P=0.80). The recalculated HR did not change significantly for OS (HR=1.58, 95%CI=1.32-1.89, P<0.01).

## DISCUSSION

### Foremost findings

The current meta-analysis included 57 English articles that incorporated 15 miRNAs and 5445 patients. As the most researched miRNA, PC patients with high blood or tissue miR-21 levels had significantly poorer OS than those with low levels. It also proved true among PC patients with high tissue miR-21 levels (multivariate analysis) and pooled analysis adjusted with the trim and fill method of OS, indicating that miR-21 is a stable and useful prognostic biomarker in PC. Moreover, a few other miRNAs had significant prognostic impact on PC, including blood miR-451a, and miR-1290 and tissue miR-10b, miR-17-5p, miR-29c, miR-126, miR-155, miR-203, miR-218, miR-221, and miR-222. Among these, blood miR-21, and miR-451a and tissue miR-23a, miR-155, and miR-218 were strong biomarkers of prognosis for PC.

### Altered expression, potential targets and pathways for studied miRNAs

In addition, an overview of the 15 miRNAs with dysregulated levels, covering the validated targets and pathways, is shown in Table 3. Most of the included miRNAs showed stable expression levels, higher or lower than the control groups except miR-200c. In brief, Table 3 could support a better understanding of the molecular biological mechanisms of miRNAs in PC.

**Table 3:** Summary of miRNAs with altered expression, their validated targets and pathways entered this study.

**miRNA**	**Reference**	**Expression**	**Potential target**	**Pathway**
10b	[35–38]	Up	None	Cell invasion
17-5p	[39–41]	Up	PTEN,RBL2	Cell cycle, invasion and proliferation
21	[4–8,43–60]	Up	BTG2,FASL,PDCD4,SPRY2	Cell apopsotis, chemoresistance, cycle, proliferation, FASL/FAS, MAPK/ERK and PI3K/AKT signaling
23a	[50, 53, 61, 62]	Up	ESRP1,FOXP2,NEDD4L	Cell invasion, epithelial-mesenchymal transition, migration and proliferation
29c	[33, 46, 69, 70]	Down	MMP2	Cell invasion, migration and Wnt signaling
126	[27, 68, 82]	Down	None	None
155	[14, 50, 51]	Up	None	None
196a	[16,17]	Up	None	None
200c	[109–111]	Unstable	None	Cell invasion and proliferation
203	[59, 112–114]	Up	None	None
218	[121–123]	Down	UGT8,VOPP1	Cell proliferation
221	[46, 50, 125, 126]	Up	None	Cell migration and proliferation
222	[28, 126, 127]	Up	NOSTRIN	None
451a	[7, 8, 23]	Up	None	None
1290	[24, 26]	Up	None	None

PTEN: phosphatase and tensin homolog; RBL2: RB transcriptional corepressor like 2; BTG2: BTG anti-proliferation factor 2; FASL: Fas ligand; PDCD4: programmed cell death 4; SPRY2: sprouty RTK signaling antagonist 2; ESRP1: epithelial splicing regulatory protein 1; FOXP2: forkhead box P2; NEDD4L: NEDD4 like E3 ubiquitin protein ligase; UGT8: UDP glycosyltransferase 8; VOPP1: VOPP1 WW domain binding protein; NOSTRIN: nitric oxide synthase trafficking; FAS: Fas cell surface death receptor; MAPK: mitogen-activated protein kinase; ERK: extracellular regulated protein kinases; PI3K: phosphoinositide-3-kinase; AKT: AKT serine/threonine kinase 1.

### Superiorities of the meta-analysis

The present work had two strengths : (1) we looked for and found out almost all studies with OS in PC patients with dysregulated miRNA levels. In addition, the recent miRNA expression pattern is shown in Tables 4 and 5 that differentiates miRNA names and the sample types. (2) The majority of included articles had large sample sizes (≥30, all but 4 studies [6, 41, 121, 125]), intensifying and widening the applicability of the prognostic outcomes for PC patients.

**Table 4:** Frequency of studies estimating prognostic value of blood miRNA expression in pancreatic cancer.

**miR**	**N**	**R**	**miR**	**N**	**R**	**miR**	**N**	**R**	**miR**	**N**	**R**
let-7b-5p	1	1	107	1	11	203	1	18	483-3p	1	6
16-2-3p	1	2	124	1	12	205	1	19	486-3p	1	24
19a-3p	1	1	125b-5p	1	13	210	1	17	602	1	2
19b-3p	1	1	150	1	10	222	1	20	629	1	25
21-5p	1	3	155	1	14	223-3p	1	1	877-5p	1	2
21	5	4-8	182	1	15	301a-3p	1	21	890	1	2
25-3p	1	1	191	1	7	373	1	22	1290	2	24,26
33a	1	9	192-5p	1	1	375	1	3	3201	1	2
34a	1	10	196a	2	16,17	451a	3	7,8,23	4525	1	8

Highlighted studies were included in the present meta-analysis; N: Number of studies estimating prognostic value; R: References.

**Table 5:** Frequency of studies estimating prognostic value of tissue miRNA expression in pancreatic cancer.

**miR**	**N**	**R**	**miR**	**N**	**R**	**miR**	**N**	**R**	**miR**	**N**	**R**	**miR**	**N**	**R**
let-7a-3	1	27	92b-3p	1	75	155	3	14,50,51	301a-3p	1	129	509-5p	1	151
let-7g*	1	28	93	1	38	181c	1	100	301b	1	38	539	1	152
let-7g	1	29	96-5p	1	76	182-5p	1	76	323-3p	1	130	545	1	153
1	1	30	100	2	50,77	183	1	101	326	1	71	548an	1	154
7-5p	1	31	101	1	78	191	1	102	328	1	68	590-5p	1	38
9-5p	1	32	103	1	79	192	2	33,103	329	1	131	613	1	155
9	1	33	107	1	80	195	1	104	337	1	132	615-5p	1	156
10a-5p	1	34	124	1	81	196a-2	1	105	342-3p	2	53,133	661	1	157
10b	4	35-38	125a-3p	1	29	196b	2	59,106	361-3p	1	134	663	1	158
15b	1	38	125a	1	68	198	2	55,107	367	1	135	664a	1	68
17-5p	3	39-41	125b	1	77	199a-3p	1	53	371-5p	1	136	664	1	159
19a	1	42	126	3	27,68,82	200c-3p	1	108	374b-5p	1	137	675-5p	1	160
21	19	5,43-60	130b	1	83	200c	3	109-111	375	1	50	675	1	28
23a	4	50,53,61,62	132	2	33,84	203	4	59,112-114	376b	1	68	708-5p	1	161
24-1	1	27	133a-1	1	27	204-5p	1	115	376c	1	68	744	1	162
25-3p	1	63	133a	2	33,85	204	1	95	377	1	138	891b	1	163
26a	1	64	135b-5p	2	86,87	205-5p	1	29	410-3p	1	139	940	1	164
27a	1	53	135b	1	88	205	2	19,116	421	1	27	1181	1	165
29a-5p	1	29	137	1	89	211	1	117	424	2	82,114	1246	1	166
29a	1	65	139-5p	1	90	212-3p	1	29	429	1	140	1247	1	167
29b-2-5p	1	66	139	1	91	212	2	28,118	448	1	141	1266	1	168
29b-3p	1	67	140	1	33	214	1	30	450b-5p	1	28	1293	1	114
29b	2	33,68	141	2	92,93	216b-5p	1	119	451	1	142	1301	1	68
29c	4	33,46,69,70	142-3p	2	53,94	216b	1	120	454	1	68	3157	1	27
30a	1	71	142-5p	1	95	217	1	50	483-3p	1	143	3613	1	68
30b	1	72	143	1	50	218	3	121-123	491	1	33	3656	1	169
30d	1	46	146a	1	28	219	1	71	494	3	144-146	4521	1	27
30e	1	27	148a*	1	28	221-3p	1	124	495	1	147	4709	1	27
31	2	50,54	148a	1	50	221	4	46,50,125,126	497	1	148	5091	1	27
34a-5p	2	29,73	148b	1	96	222	3	28,126,127	501-3p	1	149			
34a	1	46	150	1	97	223	1	128	501	1	27			
34b	1	74	153	2	98,99	224	2	46,71	506	1	150			

Highlighted studies were included in the present meta-analysis; N: Number of studies estimating prognostic value; R: References.

### Drawbacks

The following drawbacks of the current meta-analysis should considered: (1) there were numerous variables, consisting of dissimilar sample types from PC patients at different stages, cutoffs, and miRNA detection methods, among which the differences in sample type and cutoffs were the main drawbacks; (2) we only selected English articles, perhaps excluding potential papers published in other languages about PC patients with miRNA expression levels and prognostic outcomes; (3) we only chose studies estimating OS, perhaps excluding potential investigations reporting prognosis with other survival results, such as disease-free and recurrence-free survival; (4) the prognostic impact of miRNA expression levels in pancreatic cancer should be adjusted for risk factors that have an important influence on pancreatic cancer prognosis, such as age, educational level, sex, smoking, obesity, heavy alcohol intake, underlying illnesses and family history of cancer, which indicates possible mutations. However, the searched papers may not all contain the very concerned information. Therefore, the impact of bias in predicting miRNAs involved in pancreatic cancer prognosis may occur due to the lack of adjustment for risk factors in a rigorous conclusion.

### Insight for future clinical and experimental studies

Notably, this study was the first meta-analysis of the associations between abnormal miRNA levels and prognosis in PC patients. This study provides direction for further clinical and experimental study: (1) joint detection of various miRNA levels could be utilized by clinical workers and other health care providers, which might extremely expand the ability to assess the prognosis of PC patients such that immediate treatment might be supplied; (2) advances and trends regarding miRNA expression levels and the survival time of PC patients could be obviously acquired by the experimental researchers mentioned in Tables 4 and 5. In addition, miRNA molecular mechanisms could be obtained by assessing the data in Table 3; and (3) several contradictory outcomes concerning the prognostic value of miRNAs might be resolved on account of the present work.

## CONCLUSIONS

In summary, blood miR-21, miR-451a, miR-1290 and tissue miR-10b, miR-17-5p, miR-21, miR-23a, miR-29c, miR-126, miR-155, miR-203, miR-218, miR-221, miR-222 had significant prognostic value.

## MATERIALS AND METHODS

### Search strategy

**Figure 8:**
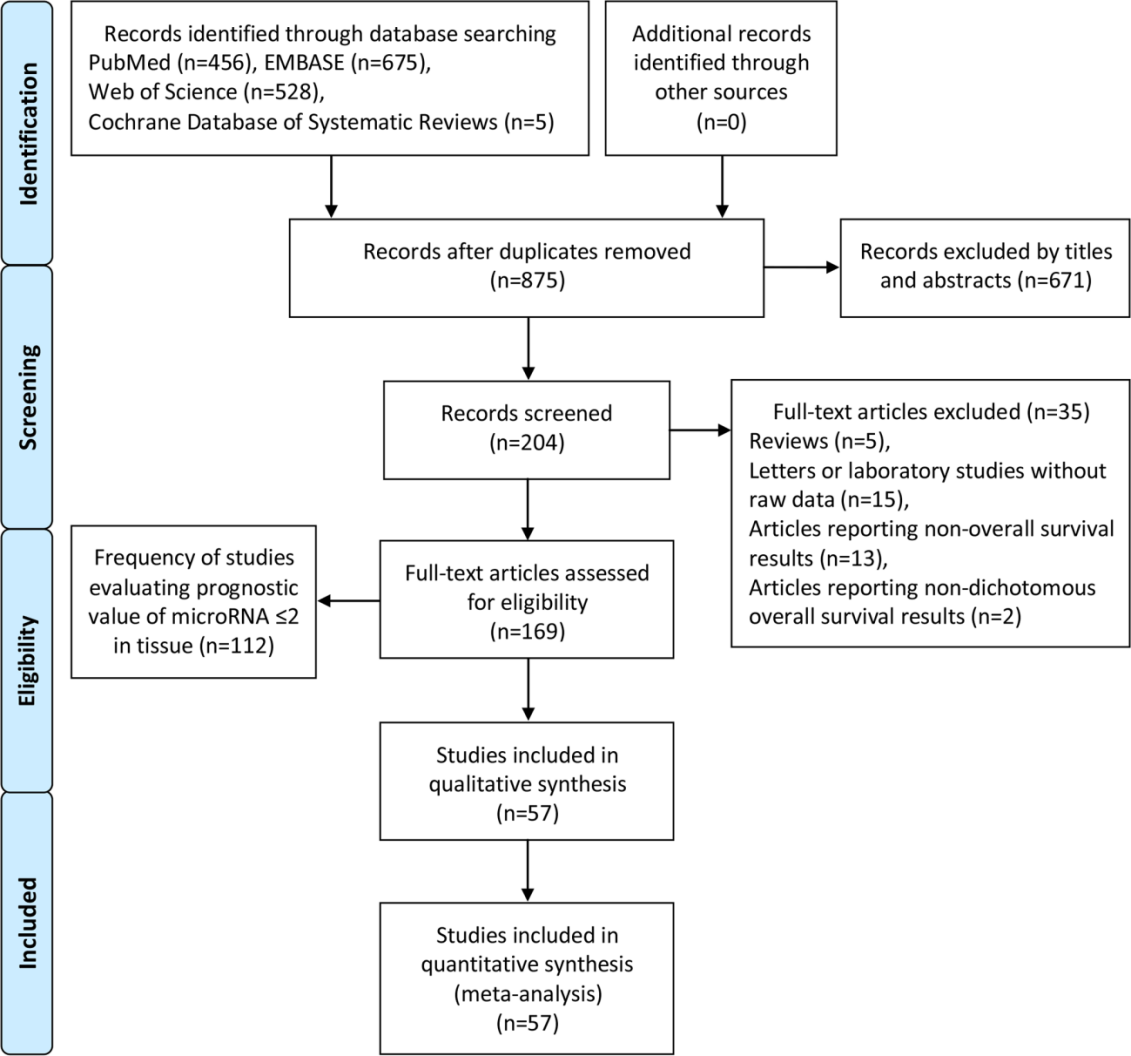
**Flow diagram of literature search and selection.**

Two independent authors (Fei Zhao and Chao Wei) performed the literature search from 4 online databases, PubMed, EMBASE, Web of Science and Cochrane Database of Systematic Reviews. Afterwards, Yue Zhang reassessed undetermined information. An extensive and comprehensive search was performed utilizing the keywords: ‘microRNA’, ‘miRNA’, ‘miR’, and ‘pancreatic cancer’, ‘pancreatic carcinoma’ and ‘pancreatic adenocarcinoma’. After duplicates were eliminated, 875 reports remained. Accordingly, 671 articles were excluded by titles and abstracts. For the residual 204 studies, 35 full-text studies were removed. The details of the literature selection are shown in Figure 8. The search deadline was June 1, 2019.

### Inclusion criteria

The inclusion criteria were as follows: (1) articles on the correlation between miRNA expression level and survival time of PC patients; (2) inclusion of estimated OS outcomes; and (3) full-text in English.

### Exclusion criteria

The exclusion criteria were as follows: (1) articles without original data (reviews, letters or laboratory studies); (2) nondichotomous miRNA level; and (3) frequency of studies evaluating OS of miRNA expression level equal or less than 2 in tissue. In addition, on the condition that more than one article was published on the same subjects, the most well-rounded paper was chosen for the present work. Likewise, if both univariate and multivariate analysis of OS were covered, the latter was chosen, as this type of analysis considers interferential factors.

### Quality assessment

Fei Zhao and Chao Wei confirmed all qualified studies that analyzed the prognostic value of miRNAs in PC, and Yue Zhang reevaluated undetermined information. Quality assessment for each paper was performed employing the modified Newcastle–Ottawa Scale (NOS) [180]. NOS scores were calculated according to selection, comparability, and outcome. Articles with NOS scores ≥6 were considered high-quality articles [181].

### Study selection

The flow chart with details of the study selection process is given in Figure 8.

### Study frequency

The frequency of studies estimating the OS of PC patients with and miRNA expressions of PC patients is presented in Tables 4 (blood) and 5 (tissue), and includes the miRNA names, the frequency of included miRNAs, and the reference number.

### Study characteristics

The fundamental particulars of the included literature are fully listed in Table 6. On the condition that the data were not offered in the article but just as Kaplan–Meier survival curves, the data were abstracted from the curves, and the generation of HR with 95% CI was next carried out employing the software Engauge Digitizer version 4.1.

**Table 6:** Characteristics of included studies about pancreatic cancer.

**miRNA**	**Study**	**Country**	**Sample**	**Number**	**Stage**	**Cut-off**	**Method**	**Follow-up (month)**	**Result**	**HR (L/H)**	**HR (H/L)**	**95%CI**
21	Liu, 2012 [4]	China	Serum	38	I-IV	Median	qRT-PCR	24	OS^u^		3.26	1.47-7.23
21	Wang, 2013 [5]	China	Serum	177	III-IV	Median	qRT-PCR	30	OS^m^		1.71	1.15-2.54
21	Abue, 2015 [6]	Japan	Plasma	24	I-IV	850	qRT-PCR	>20	OS^u^		5.99	1.95-18.40
21	Goto, 2018 [7]	Japan	Serum	32	I-IV	Median	qRT-PCR	>40	OS^u^		2.57	0.90-7.35
21	Kawamura, 2019 [8]	Japan	Plasma	55	I-II	Mean	qRT-PCR	60	OS^m^		3.10	1.19-9.10
196a	Kong, 2010 [16]	China	Serum	35	I-IV	-5.22	qRT-PCR	>16	OS^u^		3.37	1.14-9.97
196a	Yu, 2017 [17]	China	Plasma	31	None	Median	qRT-PCR	15	OS^m^		0.99	0.92-1.06
451a	Goto, 2018 [7]	Japan	Serum	32	I-IV	Median	qRT-PCR	>40	OS^u^		1.45	0.63-3.31
451a	Takahasi, 2018 [23]	Japan	Plasma	50	I-II	Median	qRT-PCR	54	OS^m^		3.20	1.07-11.94
451a	Kawamura, 2019 [8]	Japan	Plasma	55	I-II	Mean	qRT-PCR	60	OS^m^		3.60	1.13-11.31
1290	Li, 2013 [24]	USA	Serum	56	I-III	Median	qRT-PCR	>80	OS^u^		1.63	0.66-3.98
1290	Tavano, 2013 [26]	Italy	Plasma	167	I-IV	ROC	ddPCR	>40	OS^u^		1.40	1.00-1.96
10b	Nakata, 2011 [35]	Japan	FFPE	115	None	None	qRT-PCR	101	OS^u^		2.19	1.37-3.50
10b	Preis, 2011 [36]	Lebanon	FFPE	95	I-IV	5000	ISH	36	OS^u^		3.59	1.73-7.43
10b	Nguyen, 2016 [37]	USA	Frozen	55	I-II	1.5 fold	qRT-PCR	34.25	OS^u^		1.12	0.54-2.32
10b	Yang, 2017 [38]	Germany I	Frozen	69	I-IV	None	qRT-PCR	>60	OS^u^		1.99	1.07-3.73
		Germany II	Frozen	41	I-IV	None	qRT-PCR	>60	OS^u^		0.81	0.39-1.67
17-5p	Yu, 2010 [39]	Japan	FFPE	80	I-IV	5.69	qRT-PCR	100	OS^u^		1.85	1.08-3.15
17-5p	Gu, 2016 [40]	China	Tissue	58	I-IV	None	qRT-PCR	>50	OS^u^		1.89	0.98-3.64
17-5p	Zhu, 2018 [41]	China	Tissue	26	None	None	qRT-PCR	>50	OS^u^		2.18	0.77-6.17
21	Dillhoff, 2008 [43]	USA	FFPE	80	None	Median	ISH	>60	OS^u^		4.23	2.17-8.25
21	Giovannetti, 2010 [44]	Italy	Frozen	59	I-IV	Median	qRT-PCR	60.5	OS^u^		2.31	1.30-4.10
21	Hwang, 2010 [45]	Korea and Italy	Tissue	97	II-IV	Median	qRT-PCR	>60	OS^m^		3.16	1.67-6.02
21	Jamieson, 2011 [46]	UK	Frozen	48	None	Median	qRT-PCR	>50	OS^m^		3.22	1.21-8.58
21	Nagao, 2012 [47]	Japan	FFPE	65	None	Mean	qRT-PCR	>40	OS^m^		2.12	1.07-4.20
21	Caponi, 2013 [48]	Italy and UK	FFPE	57	None	Median	qRT-PCR	117.3	OS^m^		3.28	1.52-7.05
21	Kadera, 2013 [49]	USA	Tissue	145	I-II,IV	Median	ISH	100	OS^u^		1.06	0.70-1.60
21	Ma, 2013 [50]	China	Frozen	78	I-IV	2 fold	qRT-PCR	>25	OS^m^		2.60	1.15-5.87
21	Papaconstantinou, 2013 [51]	Greece	FFPE	88	None	Mean	qRT-PCR	>60	OS^m^		3.93	1.25-12.35
21	Wang, 2013 [5]	China	Tissue	65	III-IV	Median	qRT-PCR	60	OS^m^		2.24	1.14-4.37
21	Donahue, 2014 [52]	USA I	FFPE	94	I-IV	Median	ISH	72	OS^m^		1.70	1.03-2.82
		USA II	FFPE	87	I-IV	Median	ISH	72	OS^u^		0.94	0.59-1.49
21	Frampton, 2014 [53]	UK	Frozen	91	IIA,IIB	Median	qRT-PCR	>48	OS^u^		1.85	1.08-3.18
21	Mitsuhashi, 2015 [54]	Japan	FFPE	283	I-IV	75%	qRT-PCR	48	OS^u^		1.60	1.07-2.39
21	Vychytilova-Faltejskova, 2015 [55]	Czech	FFPE	74	None	27.15	qRT-PCR	>40	OS^u^		1.76	1.08-2.86
21	Morinaga, 2016 [56]	Japan	FFPE	39	None	Median	ISH	114.1	OS^u^		1.80	0.90-3.60
21	Benesova, 2018 [57]	Czech	FFPE	91	II-IV	Median	qRT-PCR	18	OS^u^		1.60	1.02-2.50
21	Xi, 2018 [58]	TCGA	Tissue	169	I-IV	Median	Downloaded	60	OS^u^		1.47	1.00-2.16
21	Zhang, 2018 [59]	GEO	Tissue	174	I-IV	Median	Downloaded	>80	OS^u^		1.89	1.37-2.62
21	Zhao, 2018 [60]	Japan	Tissue	63	0-IV	None	qRT-PCR	>60	OS^u^		2.99	1.25-7.14
23a	Ma, 2013 [50]	China	Frozen	78	I-IV	2 fold	qRT-PCR	>25	OS^u^		1.64	0.71-3.79
23a	Frampton, 2014 [53]	UK	Frozen	91	IIA,IIB	Median	qRT-PCR	>48	OS^u^		1.87	1.07-3.16
23a	Diao, 2018 [61]	China	Frozen	30	None	Median	qRT-PCR	25	OS^u^		2.55	1.10-5.92
23a	Wu, 2018 [62]	China	Tissue	52	None	3.5	qRT-PCR	>50	OS^u^		3.64	1.56-8.47
29c	Jamieson, 2011 [46]	UK	Frozen	48	None	Median	qRT-PCR	>50	OS^m^	1.89		0.68-5.26
29c	Jiang, 2015 [69]	TCGA	Frozen	132	I-IV	None	Downloaded	>50	OS^u^	1.59		1.15-2.18
29c	Zou, 2015 [70]	China	FFPE	105	I-IV	Median	qRT-PCR	30	OS^m^	1.14		1.00-1.29
29c	Wang, 2019 [33]	GEO	Tissue	178	I-IV	None	Downloaded	>80	OS^u^	1.67		1.05-2.63
126	Liang, 2018 [68]	TCGA	FFPE	175	I-IV	Median	Downloaded	>83.3	OS^m^	1.58		1.04-2.39
126	Liao, 2018 [27]	TCGA	Tissue	112	I-II	None	Downloaded	>40	OS^u^	1.51		0.98-2.32
126	Yu, 2018 [82]	TCGA	Tissue	168	I-II	Median	Downloaded	72.4	OS^m^	1.55		1.07-2.24
155	Ma, 2013 [50]	China	Frozen	78	I-IV	2 fold	qRT-PCR	>25	OS^m^		1.37	0.52-3.58
155	Papaconstantinou, 2013 [51]	Greece	FFPE	88	None	Mean	qRT-PCR	>60	OS^m^		3.14	1.09-9.09
155	Mikamori, 2017 [14]	Japan	Tissue	45	I-II	Mean	qRT-PCR	>72	OS^m^		2.63	1.07-6.46
200c	Yu, 2010 [109]	Japan	FFPE	99	I-IV	0.64	qRT-PCR	101	OS^m^	2.25		1.10-4.60
200c	Paik, 2015 [110]	Korea	FFPE	84	IB-III	0.65	qRT-PCR	140	OS^m^	0.56		0.34-0.93-
200c	Liu, 2016 [111]	China	Tissue	75	I-IV	Mean	qRT-PCR	60	OS^m^	2.31		1.73-6.38
203	Ikenaga, 2010 [112]	Japan	FFPE	107	I-IV	0.054	qRT-PCR	98	OS^m^		1.21	0.72-2.07
203	Shao, 2017 [113]	TCGA	Tissue	161	I-IV	None	Downloaded	>80	OS^u^		2.18	1.31-2.49
203	Shi, 2018 [114]	TCGA	Tissue	177	None	Median	Downloaded	>72	OS^u^		1.24	1.10-1.39
203	Zhang, 2018 [59]	GEO	Tissue	174	I-IV	Median	Downloaded	>80	OS^u^		2.27	1.57-3.27
218	Li, 2013 [121]	China	FFPE	28	None	1.5 fold	qRT-PCR	>20	OS^u^	1.86		0.80-4.35
218	Zhu, 2014 [122]	China	Frozen	113	I-IV	Mean	qRT-PCR	>50	OS^m^	2.12		1.51-2.50
218	Li, 2015 [123]	China	Frozen	107	I-IV	Median	qRT-PCR	60	OS^m^	7.24		2.01-18.28
221	Jamieson, 2011 [46]	UK	Frozen	48	None	Median	qRT-PCR	>50	OS^m^		0.92	0.34-2.54
221	Ma, 2013 [50]	China	Frozen	78	I-IV	2 fold	qRT-PCR	>25	OS^m^		2.00	0.87-4.62
221	Sarkar, 2013 [125]	USA	FFPE	24	None	None	qRT-PCR	>83.3	OS^u^		1.36	0.52-3.51
221	Wang, 2016 [126]	Germany	Frozen	37	I-II	66.7%	qRT-PCR	>40	OS^u^		2.85	1.20-6.77
222	Schultz, 2012 [28]	Denmark	FFPE	225	I-II	Median	qRT-PCR	24	OS^m^		1.39	1.06-1.84
222	Lee, 2013 [127]	China	Frozen	60	I-IV	Median	qRT-PCR	15	OS^m^		5.16	1.16-22.91
222	Wang, 2016 [126]	Germany	Frozen	37	I-II	None	qRT-PCR	>40	OS^u^		1.86	0.79-4.37

HR (L/H): hazard ratios of low expression versus high expression of miRNAs; HR (H/L): hazard ratios of high expression versus low expression of miRNAs; CI: confidence intervals; TCGA: The Cancer Genome Atlas; GEO: Gene Expression Omnibus; FFPE: formalin-fixed paraffin-embedded; qRT-PCR: quantitative real-time polymerase chain reaction; ddPCR: droplet digital polymerase chain reaction; ISH: in-situ hybridization; OS: overall survival; ^u^Univariate analysis; ^m^Multivariate analysis.

### Statistical analysis

All analyses were carried out employing Stata version 13.0 (StataCorp, College Station, TX, USA). OS was the primary and unique guideline for the prognosis of PC patients with miRNAs. The HR was regarded as significant at the P <0.05 level in case of the 95% CI not including the value 1. Furthermore, a single miRNA was considered a strong candidate if its HR was over 2. Most analyses used random-effects models other than fixed-effects models because of the dissimilarity of sample types from PC patients at dissimilar stages, cutoffs, and miRNA methods in single studies. Begg’s funnel plot was used to estimate publication bias. A two-tailed P value less than 0.05 was regarded as significant. If publication bias occurred, the trim and fill method was conducted. The sensitivity analysis was employed to assess how sensitive the entire effect size was to remove the impact of single investigations. If the point estimation was outside of the 95% CI of the entire effect value after it was excluded from the entire analysis, a single study was deemed to have undue influence.
